# A millisecond integrated quantum memory for photonic qubits

**DOI:** 10.1126/sciadv.adu5264

**Published:** 2025-03-26

**Authors:** Yu-Ping Liu, Zhong-Wen Ou, Tian-Xiang Zhu, Ming-Xu Su, Chao Liu, Yong-Jian Han, Zong-Quan Zhou, Chuan-Feng Li, Guang-Can Guo

**Affiliations:** ^1^ CAS Key Laboratory of Quantum Information, University of Science and Technology of China, Hefei 230026, China.; ^2^ Anhui Province Key Laboratory of Quantum Network, University of Science and Technology of China, Hefei 230026, China.; ^3^ CAS Center for Excellence in Quantum Information and Quantum Physics, University of Science and Technology of China, Hefei 230026, China.; ^4^ Hefei National Laboratory, University of Science and Technology of China, Hefei 230088, China.

## Abstract

Quantum memories for light are essential building blocks for quantum repeaters and quantum networks. Integrated operations of quantum memories could enable scalable application with low-power consumption. However, the photonic quantum storage lifetime in integrated devices has so far been limited to tens of microseconds, falling short of the requirements for practical applications. Here, we demonstrate quantum storage of photonic qubits for 1.021 milliseconds based on a laser-written optical waveguide fabricated in a ^151^Eu^3+^:Y_2_SiO_5_ crystal. Spin dephasing of ^151^Eu^3+^ is mitigated through dynamical decoupling applied via on-chip electric waveguides, and we obtain a storage efficiency of 12.0 ± 0.5% at 1.021 milliseconds, which is a demonstration of integrated quantum memories that outperforms the efficiency of a simple fiber delay line. Such long-lived waveguide-based quantum memory could support applications in quantum repeaters, and further combination with critical magnetic fields could enable potential application as transportable quantum memories.

## Teaser

Photonic qubits are stored in an integrated optical waveguide for 1 ms with an efficiency outperforming a fiber delay line.

## INTRODUCTION

Photonic quantum memory is a key enabler for constructing large-scale quantum networks (*1*–*4*) through approaches such as quantum repeaters (*1*, *3*, *5*, *6*) and transportable quantum memories (*7*–*11*), which have been demonstrated in various atomic platforms (*12*–*23*). As an ensemble based quantum memory (*24*–*27*), rare-earth-ion-doped crystals (REICs) have attracted much attention due to their long coherence lifetimes (*10*–*12*, *28*–*30*) and wide bandwidths (*24*, *31*, *32*). As a solid-state platform, integrated operations have been demonstrated with various fabrication techniques in REICs (*31*, *33*–*36*), enabling quantum storage in optical waveguide with high fidelity (*37*, *38*), large memory capacity (*39*, *40*), and direct telecom interface (*41*, *42*). However, the storage lifetimes of these integrated devices have been constrained to tens of microseconds (*34*, *35*) and the storage efficiency fall below that of a simple fiber delay line, posing substantial challenges for practical applications in quantum repeaters and transportable quantum memories.

In this work, we demonstrate quantum memory for light with a 1/*e* lifetime of 1.9 ms, using an integrated laser-written optical waveguide fabricated in a ^151^Eu^3+^:Y_2_SiO_5_ crystal. Leveraging the noiseless photon echo (NLPE) protocol (*43*), we achieve a quantum storage efficiency of 12.0 ± 0.05% at a storage time of 1.021 ms, far surpassing the corresponding efficiency of a fiber delay line operating in the 1550-nm C-band. A coplanar electric waveguide is used to efficiently apply the dynamical decoupling (DD) sequence, mitigating spin dephasing of the ions inside the optical waveguide (*12*, *44*).

## RESULTS

### Experiment setup

**Fig. 1.**
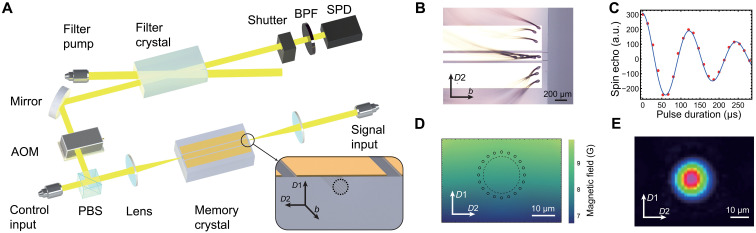
Experimental setup. (**A**) Schematic of the setup. The signal input, polarized along the *D*1 axis of the ^151^Eu^3+^:Y_2_SiO_5_ crystal, is coupled to the optical waveguide fabricated in the memory crystal. After retrieval, the beam is temporally gated by AOMs and then spectrally filtered by filter crystal and a band-pass filter (BPF). A fiber-coupled single-photon detector (SPD) lastly detects the signal. The control input, polarized along the *D*2 axis of the ^151^Eu^3+^:Y_2_SiO_5_ crystal, is coupled into the waveguide in the opposite direction and combined with the signal beam through the PBS. The control input applies the preparation sequence and optical π pulses. The filter crystal is prepared by a pump beam to create a transparent band at signal frequency. Both crystals are housed in the same cryostat, operating at a temperature of 3.2 K. The inset provides a schematic of the input surface of the memory crystal. (**B**) Microscope image of the coplanar electric waveguide fabricated on the top surface of the ^151^Eu^3+^:Y_2_SiO_5_ crystal. The waveguide is connected with multiple wires to accommodate high-power rf pulses. The electric waveguide is connected to an rf source and terminates with a 50-ohm load (not shown here). (**C**) Optically detected spin nutation measurement on the hyperfine transition between ∣±1/2〉_*g*_ and ∣±3/2〉_*g*_, achieved with a peak rf power of 4 W, demonstrating a Rabi frequency of 16.7 kHz. a.u., arbitrary units. (**D**) Simulation of the magnetic field distribution provided by the coplanar electric waveguide. The dashed circle indicates the optical mode within the optical waveguide, whereas small circles represent fabrication tracks. (**E**) Measured optical mode within the waveguide at the end surface of the crystal.

We use a 0.01% doped ^151^Eu^3+^:Y_2_SiO_5_ crystal with a size of 5 mm by 4 mm by 17 mm (*D*1 × *D*2 × *b* axes) as the substrate. This material is of particular interest due to its remarkable properties, including a spin coherence lifetime of 10 hours (*10*), coherent light storage capability for up to 1 hour (*11*), and quantum storage durations in the millisecond range (*12*, *13*). A low concentration of ^151^Eu^3+^ ions is chosen to minimize spin inhomogeneous broadening, which could otherwise limit the coherence lifetime under critical magnetic field conditions (*45*, *46*). An optical waveguide is fabricated along the *b* axis using a femtosecond-laser micromachining system (*34*, *38*, *47*, *48*). To realize the long-lived quantum storage based on DD control on the spin transitions, we have fabricated a coplanar electric waveguide (*44*, *49*) on the top surface of the crystal via lift-off technique to apply the radio frequency (rf) pulses. The magnetic field generated by the coplanar electric waveguide spatially matches well with the guide mode of the optical waveguide as illustrated in Fig. 1.

The optical waveguide comprises 20 tracks arranged along the *b* axis of Y_2_SiO_5_ crystal as shown in Fig. 1, which collectively form a circular structure to confine light (*47*, *48*). This structure can, in principle, support the transmission of arbitrary polarization modes (*38*). The absorption depth of the ^7^F_0_ → ^5^D_0_ transition for Eu^3+^ ions inside the waveguide is 2.09 and 0.38 for input light polarized along the *D*1 and *D*2 axes of the Y_2_SiO_5_ crystal, respectively. The coplanar electric waveguide, comprising three electrodes arranged along the *b* axis, is depicted in Fig. 1B. The two outer electrodes serve as the ground, whereas the middle electrode acts as the signal electrode (*50*). The electrode widths are optimized to produce a sufficiently uniform magnetic field for the ions within the optical waveguide. These electrodes are fabricated on the *D*2 × *b* surface to maximize the coupling of the magnetic field with the Eu^3+^ hyperfine transitions compared to the configuration on the *D*1 × *b* surface, see Supplementary Materials for detail.

**Fig. 2.**
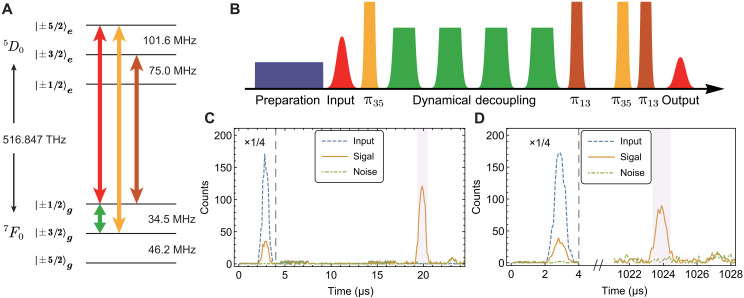
Integrated storage of single-photon level inputs for 1.021 ms. (**A**) Energy level structure of the ^7^F_0_ → ^5^D_0_ transition of ^151^Eu^3+^ ions in Y_2_SiO_5_ crystals at zero magnetic field. (**B**) Time sequence for the NLPE quantum memory with DD. After initial spectral preparation, the input pulse is absorbed by inhomogeneously broadened ^151^Eu^3+^ ions. The first π_35_ transfer the photonic excitation to spin-wave excitation. DD based on the XY4 sequence is used to rephase the spin transition. The remaining three optical π pulses rephase the optical transition, and the final echo is retrieved at the same frequency as the input. The color of each pulse corresponds to the resonance transitions, as indicated in (A). (**C** and **D**) Photon-counting histograms for NLPE memory without (C) and with DD (D), respectively. The blue dashed line represents the input, whereas the orange solid line and the green dash-dotted line represent the signal and noise, respectively, with and without inputs. Data on the left side of the gray dashed line are scaled by a factor of 1/4 for visualization. The light purple shaded region highlights the 1.1-μs detection window. With an average input photon number per pulse μ = 1.07, the SNR is 50.3 ± 16.7 for (C) and 13.1 ± 2.4 for (D).

The experimental setup is depicted in Fig. 1. The laser source (not shown) is a frequency-doubled, stabilized semiconductor laser with a center frequency of 516.847 THz and a linewidth of 0.4 kHz, resonant with ^7^F_0_ → ^5^D_0_ transition of ^151^Eu^3+^ ions (Fig. 2A). The rf signal, resonant with the hyperfine transition |± 1/2〉*_g_* → |± 3/2〉*_g_* at 34.5 MHz, is generated by an arbitrary waveform generator (Zurich HDAWG) and amplified by a 53-dB amplifier (Bruker RFA-0.1/250-150). The optical signal and control beams are generated using double-pass acousto-optic modulator (AOM). The control beam is in orthogonal polarization to that of the signal mode, allowing the polarization beam splitter (PBS) to effectively filter out coherent noise generated by the control pulses. Two single-passed AOM gates and a filter crystal are used to reduce noise in the temporal and frequency domains, respectively. The filter crystal, a 0.1% doped ^151^Eu^3+^:Y_2_SiO_5_ with a length of 30 mm, provides a typical absorption depth of 9.1 with a 2-MHz transparent window centered at the signal frequency. Both the memory crystal and the filter crystal are maintained at 3.2 K using a closed-cycle cryostat (Montana Instruments).

The coplanar electric waveguide and the cables inside the cryostat together exhibit a reflection of −25 dB and a transmission of −0.3 dB at the operating frequency of 34.5 MHz. This integrated configuration achieves a Rabi frequency of Ω = 16.7 kHz for the hyperfine transition | ± 1/2〉*_g_* → | ± 3/2〉*_g_* at an input peak power of 4 W (Fig. 1C). The coplanar electric waveguide facilitates efficient spin driving with straightforward integration, substantially reducing the required rf power compared to conventional bulk rf coils (*11*, *12*). For comparison, we also apply an rf field using bulk coils within the same system. The coil, with a length matching that of the crystal, consists of 15 turns and has a radius of 10 mm. Achieving the same Rabi frequency requires a power input of 330 W, which is 82 times higher than that of the electric waveguide. The Rabi frequency can be further increased by narrowing the electrode width, albeit at the cost of increased rf field inhomogeneity, see Supplementary Materials for details. In this work, we select an electrode width of 150 μm to strike a balance between minimizing the required rf power and maintaining acceptable field homogeneity.

### Storage of single-photon level inputs

The ^7^F_0_ → ^5^D_0_ transition of ^151^Eu^3+^ is a strictly forbidden transition and becomes weakly allowed in Y_2_SiO_5_ crystals. Given the low sample absorption depth (~1.5), we use the NLPE protocol for spin-wave quantum storage. This approach can fully use the original sample absorption to capture input photons, making it particularly well suited to the current sample (*34*, *43*, *51*). The experimental time sequence is illustrated in Fig. 2B. An absorption band of 1.8 MHz is prepared within a 4-MHz transparent window on the |± 1/2〉*_g_* → |± 5/2〉*_e_* transition (fig. S2B). The input signals are weak coherent pulses with an average photon number per pulse μ = 1.07. The Gaussian-shaped pulses have a full width at half maximum of 0.8 μs and a total duration of 1.7 μs. Four optical π pulses are required to implement the NLPE memory where π_13_ pulses drive the transition |± 1/2〉*_g_* → |± 3/2〉*_e_* and π_35_ pulses drive |± 3/2〉*_g_* → | ± 5/2〉*_e_*. We use adiabatic π pulses with a bandwidth of 2.2 MHz and a duration of 1.5 μs to drive the optical transition. The peak power is 50 mW for π_35_ and 55 mW for π_13_, respectively, corresponding to a same Rabi frequency of 1.2 MHz. In bulk demonstrations, achieving the same Rabi frequency would require six times more power (*43*). The final echo is emitted at the same frequency as that of the input signal (*34*). As shown in Fig. 2C, the NLPE storage efficiency is 17.8 ± 0.9% with a detection window of 1.1 μs at a storage time of 17 μs. The noise per temporal mode *p_N_* = 0.38 ± 0.13%, and the signal-to-noise ratio (SNR) is 50.7 ± 16.7.

When an input photon is stored as a collective spin-wave excitation of an atomic ensemble, the spin-wave excitation dephases within tens of microseconds due to spin inhomogeneous broadening on the order of tens of kilohertz (*34*). In our system, the 1/*e* spin-wave storage lifetime of NLPE memory is ~42 μs (fig. S3B). To overcome this limit, we use the DD to rephase the spin transitions and extend the storage lifetime to the spin coherence lifetime. Previously, this technique has only been demonstrated with atomic frequency comb (AFC) quantum memories in bulk crystals (*12*, *13*). In our experiment, the DD sequence is applied between the first π_35_ and the π_13_ pulse, to rephase the spin transition between |± 1/2〉*_g_* and |± 3/2〉*_g_*. We use the XY4 sequence (X-Y-X-Y), which is designed to preserve arbitrary initial states. Adiabatic pulses are used in the sequence, offering better control over spin transitions. These pulses produce a flatter spectrum in the frequency domain compared to square pulses, ensuring consistent driving across the entire bandwidth (*12*, *13*). The lifetime of the NLPE with DD (NLPE-DD) is extended to 1.90 ± 0.04 ms (fig. S5C). At a storage time of 1.021 ms, the storage efficiency is 12.0 ± 0.5% for a detection window of 1.1 μs (Fig. 2D). The noise per temporal mode *p_N_* = 0.98 ± 0.15%, resulting in an SNR of 13.1 ± 2.4.

The noise notably increases after introducing DD into NLPE, primarily due to imperfections in the rf π pulse within the DD sequence. Imperfect π pulses reduce storage efficiency and leave residual population in ∣±3/2〉_*g*_ after the DD process. Subsequently, the optical π_35_ pulse transfers this residual population to ∣±5/2〉_*e*_, resulting in indistinguishable spontaneous emission noise. By comparing the NLPE storage efficiencies with and without DD, we could estimate that the XY4 sequence leaves 0.3 ± 0.2% population in ∣±3/2〉_*g*_ in this device (see Supplementary Materials for details). This issue can be mitigated by extending the width of the middle electrode to improve the quality of DD pulses across the ensemble, albeit at the cost of increased heating (fig. S5). Our implementation of NLPE-DD in a waveguide achieves an SNR approximately twice that of previous demonstrations of AFC memories with DD in bulk crystals (*12*, *13*). The noise levels in both our experiments and previous studies are approximately the same, and the enhanced SNR in our work is primarily due to the improved storage efficiency of the NLPE protocol, despite a sample with a lower concentration of Eu^3+^ ions is used in the current work.

### Storage of time-bin qubits

To benchmark the quantum storage performances of the current device, we further encode time-bin qubits on the input pulses. Four states are prepared as input qubits: ∣*e*〉, ∣*l*〉, ∣*e*〉 + ∣*l*〉, and ∣*e*〉 + *i*∣*l*〉, where ∣*e*〉 and ∣*l*〉 represent the early bin and the late bin, respectively. Each bin has a duration of 1.7 μs, with a 2-μs separation between the two bins. For superposition states ∣*e*〉 + ∣*l*〉 and ∣*e*〉 + *i*∣*l*〉, an unbalanced Mach-Zehnder interferometer (MZI) is typically required for analysis. Here, we use the NLPE memory itself as an unbalanced MZI (*34*, *43*) for analysis by splitting the latter π_13_ pulse into two ${\left(\frac{\pi }{2}\right)}_{13}$ pulses separated by 2 μs. The two input pulses resulted in three output pulses, enabling analysis of the superposition states by observing constructive and destructive interference in the central bin of the output. This interference was controlled by applying a phase shift between the two ${\left(\frac{\pi }{2}\right)}_{13}$ pulses.

**Fig. 3.**
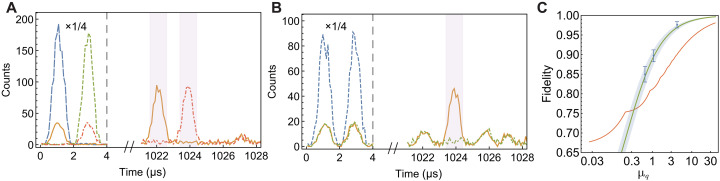
Integrated storage of time-bin qubits for 1.021 ms. (**A**) Photon-counting histogram for input qubits of ∣*e*〉 and ∣*l*〉, with an average input photon number per qubit μ*_q_* = 1.07. The inputs are shown with blue (∣*e*〉) and green (∣*l*〉) dashed lines. The outputs for ∣*e*〉 and ∣*l*〉 are shown as orange solid and red dash-dotted lines, respectively. The light purple shaded area highlights the detection windows, whereas data on the left of the gray dashed line are scaled by a factor of 1/4 for visualization, for (A) and (B). (**B**) Photon-counting histogram for measurements of ∣*e*〉 + ∣*l*〉 states with μ*_q_* = 1.07. The blue dashed line represents the input, whereas the orange solid and green dash-dotted lines correspond to measurements with constructive and destructive interference, respectively. (**C**) Total fidelity of the memory as a function of the input photon number per qubit μ_*q*_. The red solid line indicates the classical bound, which is the maximum fidelity achievable by any classical device considering the storage efficiency of 12.0% and the statistics of input coherent states (*21*). The blue dots represent the measured storage fidelity (see Table 1), whereas the green line shows the theoretical fidelity calculated based on the measured noise and storage efficiency. Error bars correspond to 1 SD. Details of the fidelity calculation are provided in the Supplementary Materials.

Figure 3 (A and B) provides photon counting histograms for input qubits of ∣*e*〉, ∣*l*〉, and ∣*e*〉 + ∣*l*〉, with an average input photon number per qubit (in two temporal modes) μ*_q_* = 1.07. Histograms for measurements for ∣*e*〉 + *i*∣*l*〉 are provided in fig. S3C. The measured total fidelity *F*_*T*_ = 89.7 ± 1.5%. Here, $F_{T}=\frac{1}{3}\left(F_{|e\rangle }+F_{|l\rangle }\right)/2+\frac{2}{3}\left(F_{|e\rangle +|l\rangle }+F_{|e\rangle +i|l\rangle }\right)/2$, where *F*_∣*i*〉_ denotes the storage fidelity of the input qubit ∣*i*〉 (*11*, *34*, *52*). Additional measurements of storage fidelity are performed for various input photon levels (Table 1). The total fidelity improves to 97.7 ± 0.8% with μ_*q*_ = 4.21 and decreases to 86.1 ± 2.0% with μ_*q*_ = 0.66. As shown in Fig. 3C, all measured fidelities exceeds the maximal fidelity achievable using a classical “measure-and-prepare” strategy, considering the finite efficiency and the statistics of input coherent states (*21*, *34*, *52*). This unambiguously demonstrates that the long-lived integrated quantum memory operates within the quantum regime.

**Table 1.** Storage fidelity with various input levels. μ_*q*_ is the average photon number per qubit. *F*_∣*e*〉_ is the fidelity for input qubit ∣*e*〉, and the definition is similar for *F*_∣*l*〉_, *F*_∣*e*〉+∣*l*〉_, and *F*_∣*e*〉+*i*∣*l*〉_. *F*_*T*_ is the total fidelity.

μ_*q*_	*F*_∣*e*〉_	*F*_∣*l*〉_	*F*_∣*e*〉∣*l*〉_	*F*_∣*e*〉+*i*∣*l*〉_	*F*_*T*_
0.66	90.7 ± 1.3%	90.8 ± 1.4%	84.2 ± 2.3%	83.3 ± 2.3%	86.1 ± 2.0%
1.07	92.4 ± 1.0%	93.8 ± 0.9%	87.7 ± 1.7%	88.3 ± 1.7%	89.7 ± 1.5%
4.21	98.6 ± 0.5%	98.2 ± 0.5%	97.5 ± 0.9%	97.2 ± 0.9%	97.7 ± 0.8%

To create a practically competitive device, an intuitive and important target would be beating optical fiber delay line, specifically achieving a storage efficiency greater than the transmission efficiency of a telecom fiber for the same storage duration. The storage efficiency of the current device is 12.0 ± 0.5% at 1.021 ms, outperforming the transmission efficiency of a fiber delay line by more than three orders of magnitude. The storage time is two orders longer than current photonic quantum memories in integrated devices (see fig. S4 for an overview), laying out the foundation for practical applications of integrated quantum memories in large-scale quantum networks (*36*, *53*).

## DISCUSSION

The short storage time of integrated quantum memories (*31*, *33*, *35*–*37*) has been a substantial obstacle to their practical applications in long-distance quantum networks (*1*–*5*, *53*). Here, we demonstrate a 1.021-ms integrated quantum memory for photonic qubits, based on a laser-written optical waveguide combined with DD through coplanar electric waveguide. This storage time is sufficient to support the round-trip communication between quantum repeater nodes separated by 100 km (*3*, *24*). The integration of optical waveguides and coplanar electric waveguides enables precise and efficient manipulations of hyperfine transitions of rare-earth dopants. The resulting performance, including fidelity and efficiency, is competitive compared to similar implementations in bulk crystals (*12*, *13*). This device could support multiplexed operations, primarily in the time and frequency domains, to increase its multimode capacity, whereas in the spatial domain, the multimode capacity is currently constrained by challenges in confining rf fields. The demonstrated storage efficiency of 12.0 ± 0.5% surpasses the transmission efficiency of a fiber delay line and further enhancing the efficiency to unity could be achieved by using impedance-matched optical cavities (*54*, *55*). In addition, the use of a low-concentration sample with small spin inhomogeneous broadening suggests the potential for extending storage times to the order of minutes by operating in critical magnetic fields (*9*, *11*). Such advancements could unlock new opportunities for transportable quantum memories (*5*, *6*, *11*).

## MATERIALS AND METHODS

The optical waveguide is fabricated by a femtosecond laser micromachining system (WOPhotonics, Lithuania). The femtosecond laser is set to 1030 nm with 201.9-kHz repetition rate and 210-fs pulse duration. During fabrication, the laser is polarized along the *D*2 axis of the ^151^Eu^3+^:Y_2_SiO_5_ crystal and projects along the *D*1 axis of the ^151^Eu^3+^:Y_2_SiO_5_ crystal. The laser is focused by a ×100 objective with numerical aperture = 0.7. The laser moves along the *b* axis to form a notch. We constructed a cylinder with 20 notches with radius *r* = 20 μm and centered 15 μm under the top surface of the crystal. To compensate the power reduction of focus point by depth, the laser power changes gradiently along the *D*1 axis, from 68 nJ for deepest to 64 nJ for shallowest, resulting in consistent notches. This symmetrical structure supports all polarization (*38*, *48*). The free-space coupling efficiency from control input to signal input (as shown in Fig. 1A) is 50% for light polarized along the *D*1 axis and 25% for polarization along the *D*2 axis respectively. The optical path transmission efficiency without the waveguide is ~65%. We obtain that the insertion loss is 1.1 dB for *D*1 axis polarization and 4.1 dB for *D*2 axis polarization for the device. The coplanar electric waveguide is manufactured by lift-off technique after the fabrication of optical waveguide. We use ultraviolet lithography (Karl Suss, MABA6) on photoresist (NR9-3000PY) to form a 1-μm-thick pattern and then use electron beam evaporation (K. J. Lesker, LAB 18) to coat 50-nm Ti, 720-nm Cu, and 30-nm Au onto the crystal to form the electric waveguide.

## Supplementary Materials

### This PDF file includes:

Supplementary Text Figs. S1 to S5 References

## References

[1] H.-J. Briegel, W. Dür, J. I. Cirac, P. Zoller, Quantum repeaters: The role of imperfect local operations in quantum communication. Phys. Rev. Lett. 81, 5932–5935 (1998).

[2] L.-M. Duan, M. D. Lukin, J. I. Cirac, P. Zoller, Long-distance quantum communication with atomic ensembles and linear optics. Nature 414, 413–418 (2001). 11719796 10.1038/35106500

[3] N. Sangouard, C. Simon, H. De Riedmatten, N. Gisin, Quantum repeaters based on atomic ensembles and linear optics. Rev. Mod. Phys. 83, 33 (2011).

[4] A. I. Lvovsky, B. C. Sanders, W. Tittel, Optical quantum memory. Nat. Photonics 3, 706–714 (2009).

[5] M. Gündoğan, J. S. Sidhu, V. Henderson, L. Mazzarella, J. Wolters, D. K. L. Oi, M. Krutzik, Proposal for space-borne quantum memories for global quantum networking. npj Quantum Inf. 7, 128 (2021).

[6] M. Gündoǧan, J. S. Sidhu, M. Krutzik, D. K. L. Oi, Time-delayed single satellite quantum repeater node for global quantum communications. Opt. Quantum 2, 140–147 (2024).

[7] S. E. Wittig, S. M. Wittig, A. Berquanda, M. Zhong, M. Sellars, Concept for single-satellite global quantum key distribution using a solid state quantum memory, in *68th International Astronautical Congress (IAC)* (IAC-17, International Astronautical Federation, 2017), vol. 8, p. 5040–5050.

[8] J. Bland-Hawthorn, M. J. Sellars, J. G. Bartholomew, Quantum memories and the double-slit experiment: Implications for astronomical interferometry. J. Opt. Soc. Am. B. 38, A86–A98 (2021).

[9] M. Zhong, M. P. Hedges, R. L. Ahlefeldt, J. G. Bartholomew, S. E. Beavan, S. M. Wittig, J. J. Longdell, M. J. Sellars, Optically addressable nuclear spins in a solid with a six-hour coherence time. Nature 517, 177–180 (2015). 25567283 10.1038/nature14025

[10] F. Wang, M. Ren, W. Sun, M. Guo, M. J. Sellars, R. L. Ahlefeldt, J. G. Bartholomew, J. Yao, S. Liu, M. Zhong, Nuclear spins in a solid exceeding 10-hour coherence times for ultra-long-term quantum storage. PRX Quantum 6, 010302 (2025).

[11] Y. Ma, Y.-Z. Ma, Z.-Q. Zhou, C.-F. Li, G.-C. Guo, One-hour coherent optical storage in an atomic frequency comb memory. Nat. Commun. 12, 2381 (2021). 33888720 10.1038/s41467-021-22706-y PMC8062444

[12] A. Ortu, A. Holzäpfel, J. Etesse, M. Afzelius, Storage of photonic time-bin qubits for up to 20 ms in a rare-earth doped crystal. npj Quantum Inf. 8, 29 (2022).

[13] P. Jobez, C. Laplane, N. Timoney, N. Gisin, A. Ferrier, P. Goldner, M. Afzelius, Coherent spin control at the quantum level in an ensemble-based optical memory. Phys. Rev. Lett. 114, 230502 (2015). 26196785 10.1103/PhysRevLett.114.230502

[14] A. J. Stolk, K. L. van der Enden, M. C. Slater, I. te Raa-Derckx, P. Botma, J. van Rantwijk, J. J. B. Biemond, R. A. J. Hagen, R. W. Herfst, W. D. Koek, A. J. H. Meskers, R. Vollmer, E. J. van Zwet, M. Markham, A. M. Edmonds, J. F. Geus, F. Elsen, B. Jungbluth, C. Haefner, C. Tresp, J. Stuhler, S. Ritter, R. Hanson, Metropolitan-scale heralded entanglement of solid-state qubits. Sci. Adv. 10, eadp6442 (2024). 39475617 10.1126/sciadv.adp6442 PMC11524177

[15] J.-L. Liu, X. Y. Luo, Y. Yu, C.-Y. Wang, B. Wang, Y. Hu, J. Li, M.-Y. Zheng, B. Yao, Z. Yan, D. Teng, J.-W. Jiang, X.-B. Liu, X.-P. Xie, J. Zhang, Q.-H. Mao, X. Jiang, Q. Zhang, X.-H. Bao, J.-W. Pan, Creation of memory–memory entanglement in a metropolitan quantum network. Nature 629, 579–585 (2024). 38750235 10.1038/s41586-024-07308-0

[16] C. M. Knaut, A. Suleymanzade, Y.-C. Wei, D. R. Assumpcao, P.-J. Stas, Y. Q. Huan, B. Machielse, E. N. Knall, M. Sutula, G. Baranes, N. Sinclair, C. De-Eknamkul, D. S. Levonian, M. K. Bhaskar, H. Park, M. Lončar, M. D. Lukin, Entanglement of nanophotonic quantum memory nodes in a telecom network. Nature 629, 573–578 (2024). 38750231 10.1038/s41586-024-07252-z PMC11096112

[17] L.-M. Duan, C. Monroe, *Colloquium*: Quantum networks with trapped ions. Rev. Mod. Phys. 82, 1209–1224 (2010).

[18] V. Krutyanskiy, M. Canteri, M. Meraner, J. Bate, V. Krcmarsky, J. Schupp, N. Sangouard, B. P. Lanyon, Telecom-wavelength quantum repeater node based on a trapped-ion processor. Phys. Rev. Lett. 130, 213601 (2023). 37295084 10.1103/PhysRevLett.130.213601

[19] T. van Leent, M. Bock, F. Fertig, R. Garthoff, S. Eppelt, Y. Zhou, P. Malik, M. Seubert, T. Bauer, W. Rosenfeld, W. Zhang, C. Becher, H. Weinfurter, Entangling single atoms over 33 km telecom fibre. Nature 607, 69–73 (2022). 35794269 10.1038/s41586-022-04764-4 PMC9259499

[20] B. Hensen, H. Bernien, A. E. Dréau, A. Reiserer, N. Kalb, M. S. Blok, J. Ruitenberg, R. F. Vermeulen, R. N. Schouten, C. Abellán, W. Amaya, V. Pruneri, M. W. Mitchell, M. Markham, D. J. Twitchen, D. Elkouss, S. Wehner, T. H. Taminiau, R. Hanson, Loophole-free Bell inequality violation using electron spins separated by 1.3 kilometres. Nature 526, 682–686 (2015). 26503041 10.1038/nature15759

[21] H. P. Specht, C. Nölleke, A. Reiserer, M. Uphoff, E. Figueroa, S. Ritter, G. Rempe, A single-atom quantum memory. Nature 473, 190–193 (2011). 21532588 10.1038/nature09997

[22] M. D. Eisaman, A. André, F. Massou, M. Fleischhauer, A. S. Zibrov, M. D. Lukin, Electromagnetically induced transparency with tunable single-photon pulses. Nature 438, 837–841 (2005). 16341010 10.1038/nature04327

[23] S. Ritter, C. Nölleke, C. Hahn, A. Reiserer, A. Neuzner, M. Uphoff, M. Mücke, E. Figueroa, J. Bochmann, G. Rempe, An elementary quantum network of single atoms in optical cavities. Nature 484, 195–200 (2012). 22498625 10.1038/nature11023

[24] X. Liu, J. Hu, Z. F. Li, X. Li, P. Y. Li, P. J. Liang, Z. Q. Zhou, C. F. Li, G. C. Guo, Heralded entanglement distribution between two absorptive quantum memories. Nature 594, 41–45 (2021). 34079139 10.1038/s41586-021-03505-3

[25] D. Lago-Rivera, S. Grandi, J. V. Rakonjac, A. Seri, H. de Riedmatten, Telecom-heralded entanglement between multimode solid-state quantum memories. Nature 594, 37–40 (2021). 34079135 10.1038/s41586-021-03481-8

[26] H. de Riedmatten, M. Afzelius, M. U. Staudt, C. Simon, N. Gisin, A solid-state light–matter interface at the single-photon level. Nature 456, 773–777 (2008). 19079056 10.1038/nature07607

[27] M. P. Hedges, J. J. Longdell, Y. Li, M. J. Sellars, Efficient quantum memory for light. Nature 465, 1052–1056 (2010). 20577210 10.1038/nature09081

[28] T. Böttger, C. Thiel, R. Cone, Y. Sun, Effects of magnetic field orientation on optical decoherence in Er^3+^:Y_2_SiO_5_. Phys. Rev. B.Condens. Matter Mater. Phys. 79, 115104 (2009).

[29] M. Rančić, M. P. Hedges, R. L. Ahlefeldt, M. J. Sellars, Coherence time of over a second in a telecom-compatible quantum memory storage material. Nat. Phys. 14, 50–54 (2018).

[30] G. Heinze, C. Hubrich, T. Halfmann, Stopped light and image storage by electromagnetically induced transparency up to the regime of one minute. Phys. Rev. Lett. 111, 033601 (2013). 23909316 10.1103/PhysRevLett.111.033601

[31] E. Saglamyurek, N. Sinclair, J. Jin, J. A. Slater, D. Oblak, F. Bussières, M. George, R. Ricken, W. Sohler, W. Tittel, Broadband waveguide quantum memory for entangled photons. Nature 469, 512–515 (2011). 21228775 10.1038/nature09719

[32] E. Saglamyurek, M. Grimau Puigibert, Q. Zhou, L. Giner, F. Marsili, V. B. Verma, S. Woo Nam, L. Oesterling, D. Nippa, D. Oblak, W. Tittel, A multiplexed light-matter interface for fibre-based quantum networks. Nat. Commun. 7, 11202 (2016). 27046076 10.1038/ncomms11202 PMC4822043

[33] T. Zhong, J. M. Kindem, J. G. Bartholomew, J. Rochman, I. Craiciu, E. Miyazono, M. Bettinelli, E. Cavalli, V. Verma, S. W. Nam, F. Marsili, M. D. Shaw, A. D. Beyer, A. Faraon, Nanophotonic rare-earth quantum memory with optically controlled retrieval. Science 357, 1392–1395 (2017). 28860208 10.1126/science.aan5959

[34] T.-X. Zhu, M. X. Su, C. Liu, Y. P. Liu, C. F. Wang, P. X. Liu, Y. J. Han, Z. Q. Zhou, C. F. Li, G. C. Guo, Integrated spin-wave quantum memory. Natl. Sci. Rev. 11, nwae161 (2024). 39440262 10.1093/nsr/nwae161 PMC11493096

[35] J. V. Rakonjac, G. Corrielli, D. Lago-Rivera, A. Seri, M. Mazzera, S. Grandi, R. Osellame, H. de Riedmatten, Storage and analysis of light-matter entanglement in a fiber-integrated system. Sci. Adv. 8, eabn3919 (2022). 35857480 10.1126/sciadv.abn3919 PMC9714774

[36] Z.-Q. Zhou, C. Liu, C.-F. Li, G.-C. Guo, D. Oblak, M. Lei, A. Faraon, M. Mazzera, H. de Riedmatten, Photonic integrated quantum memory in rare-earth doped solids. Laser Photonics Rev. 17, 2300257 (2023).

[37] C. Liu, T. X. Zhu, M. X. Su, Y. Z. Ma, Z. Q. Zhou, C. F. Li, G. C. Guo, On-demand quantum storage of photonic qubits in an on-chip waveguide. Phys. Rev. Lett. 125, 260504 (2020). 33449731 10.1103/PhysRevLett.125.260504

[38] T.-X. Zhu, C. Liu, M. Jin, M. X. Su, Y. P. Liu, W. J. Li, Y. Ye, Z. Q. Zhou, C. F. Li, G. C. Guo, On-demand integrated quantum memory for polarization qubits. Phys. Rev. Lett. 128, 180501 (2022). 35594095 10.1103/PhysRevLett.128.180501

[39] A. Seri, D. Lago-Rivera, A. Lenhard, G. Corrielli, R. Osellame, M. Mazzera, H. de Riedmatten, Quantum storage of frequency-multiplexed heralded single photons. Phys. Rev. Lett. 123, 080502 (2019). 31491206 10.1103/PhysRevLett.123.080502

[40] N. Sinclair, E. Saglamyurek, H. Mallahzadeh, J. A. Slater, M. George, R. Ricken, M. P. Hedges, D. Oblak, C. Simon, W. Sohler, W. Tittel, Spectral multiplexing for scalable quantum photonics using an atomic frequency comb quantum memory and feed-forward control. Phys. Rev. Lett. 113, 053603 (2014). 25126920 10.1103/PhysRevLett.113.053603

[41] D.-C. Liu, P. Y. Li, T. X. Zhu, L. Zheng, J. Y. Huang, Z. Q. Zhou, C. F. Li, G. C. Guo, On-demand storage of photonic qubits at telecom wavelengths. Phys. Rev. Lett. 129, 210501 (2022). 36461974 10.1103/PhysRevLett.129.210501

[42] M. F. Askarani, M. G. Puigibert, T. Lutz, V. B. Verma, M. D. Shaw, S. W. Nam, N. Sinclair, D. Oblak, W. Tittel, Storage and reemission of heralded telecommunication-wavelength photons using a crystal waveguide. Phys. Rev. Appl. 11, 054056 (2019).

[43] Y.-Z. Ma, M. Jin, D. L. Chen, Z. Q. Zhou, C. F. Li, G. C. Guo, Elimination of noise in optically rephased photon echoes. Nat. Commun. 12, 4378 (2021). 34282136 10.1038/s41467-021-24679-4 PMC8289862

[44] Y. Wiemann, J. Simmendinger, C. Clauss, L. Bogani, D. Bothner, D. Koelle, R. Kleiner, M. Dressel, M. Scheffler, Observing electron spin resonance between 0.1 and 67 GHz at temperatures between 50 mK and 300 K using broadband metallic coplanar waveguides. Appl. Phys. Lett. 106, 193505 (2015).

[45] Y.-Z. Ma, Y.-C. Lv, T.-S. Yang, Y. Ma, Z.-Q. Zhou, C.-F. Li, G.-C. Guo, Monte Carlo simulation of the nuclear spin decoherence process in Eu^3+^:Y_2_ SiO_5_ crystals. Phys. Rev. B. 107, 014310 (2023).

[46] P.-J. Liang, T.-X. Zhu, Y.-X. Xiao, Y.-Y. Wang, Y.-J. Han, Z.-Q. Zhou, C.-F. Li, Concentration-dependent optical and spin inhomogeneous linewidth of europium-doped yttrium orthosilicate crystals. Acta Phys. Sin. 73, 100301 (2024).

[47] A. G. Okhrimchuk, A. V. Shestakov, I. Khrushchev, J. Mitchell, Depressed cladding, buried waveguide laser formed in a YAG:Nd^3+^ crystal by femtosecond laser writing. Opt. Lett. 30, 2248–2250 (2005). 16190433 10.1364/ol.30.002248

[48] N. Skryabin, A. Kalinkin, I. Dyakonov, S. Kulik, Femtosecond laser written depressed-cladding waveguide 2 × 2, 1 × 2 and 3 × 3 directional couplers in Tm^3+^:YAG crystal. Micromachines 11, 1 (2020). 10.3390/mi11010001 PMC7019769 31861295

[49] R. N. Simons, *Coplanar Waveguide Circuits, Components, and Systems* (John Wiley & Sons, 2004).

[50] C. P. Wen, Coplanar waveguide: A surface strip transmission line suitable for nonreciprocal gyromagnetic device applications. IEEE Trans. Microw. Theory Tech. 17, 1087–1090 (1969).

[51] M. Jin, Y.-Z. Ma, Z.-Q. Zhou, C.-F. Li, G.-C. Guo, A faithful solid-state spin-wave quantum memory for polarization qubits. Sci. Bull. 67, 676–678 (2022). 10.1016/j.scib.2022.01.019 36546130

[52] M. Gündoğan, P. M. Ledingham, K. Kutluer, M. Mazzera, H. de Riedmatten, Solid state spin-wave quantum memory for time-bin qubits. Phys. Rev. Lett. 114, 230501 (2015). 26196784 10.1103/PhysRevLett.114.230501

[53] X. Liu, X.-M. Hu, T.-X. Zhu, C. Zhang, Y.-X. Xiao, J.-L. Miao, Z.-W. Ou, P.-Y. Li, B.-H. Liu, Z.-Q. Zhou, C.-F. Li, G.-C. Guo, Distributed quantum computing over 7.0 km. Nat. Commun. 15, 8529 (2024). 39358375 10.1038/s41467-024-52912-3 PMC11447119

[54] P. Jobez, I. Usmani, N. Timoney, C. Laplane, N. Gisin, M. Afzelius, Cavity-enhanced storage in an optical spin-wave memory. New J. Phys. 16, 083005 (2014).

[55] M. Afzelius, C. Simon, Impedance-matched cavity quantum memory. Phys. Rev. A 82, 022310 (2010).

[56] A. Stoneham, Shapes of inhomogeneously broadened resonance lines in solids. Rev. Mod. Phys. 41, 82 (1969).

[57] M. Gündoğan, P. M. Ledingham, A. Almasi, M. Cristiani, H. de Riedmatten, Quantum storage of a photonic polarization qubit in a solid. Phys. Rev. Lett. 108, 190504 (2012). 23003015 10.1103/PhysRevLett.108.190504

[58] M. Curty, N. Lütkenhaus, Intercept-resend attacks in the Bennett-Brassard 1984 quantum-key-distribution protocol with weak coherent pulses. Phys. Rev. A 71, 062301 (2005).

[59] I. Roos, K. Mølmer, Quantum computing with an inhomogeneously broadened ensemble of ions: Suppression of errors from detuning variations by specially adapted pulses and coherent population trapping. Phys. Rev. A 69, 022321 (2004).

[60] V. Damon, M. Bonarota, A. Louchet-Chauvet, T. Chaneliere, J.-L. Le Gouët, Revival of silenced echo and quantum memory for light. New J. Phys. 13, 093031 (2011).

[61] E. Zambrini Cruzeiro, F. Fröwis, N. Timoney, M. Afzelius, Noise in optical quantum memories based on dynamical decoupling of spin states. J. Mod. Opt. 63, 2101–2113 (2016).

[62] F. Könz, Y. Sun, C. W. Thiel, R. L. Cone, R. W. Equall, R. L. Hutcheson, R. M. Macfarlane, Temperature and concentration dependence of optical dephasing, spectral-hole lifetime, and anisotropic absorption in Eu^3+^:Y_2_SiO_5_. Phys. Rev. B 68, 085109 (2003).

[63] E. Saglamyurek, N. Sinclair, J. Jin, J. A. Slater, D. Oblak, F. Bussières, M. George, R. Ricken, W. Sohler, W. Tittel, Conditional detection of pure quantum states of light after storage in a Tm-doped waveguide. Phys. Rev. Lett. 108, 083602 (2012). 22463529 10.1103/PhysRevLett.108.083602

[64] J. Jin, J. A. Slater, E. Saglamyurek, N. Sinclair, M. George, R. Ricken, D. Oblak, W. Sohler, W. Tittel, Two-photon interference of weak coherent laser pulses recalled from separate solid-state quantum memories. Nat. Commun. 4, 2386 (2013). 23985479 10.1038/ncomms3386

[65] I. Craiciu, M. Lei, J. Rochman, J. M. Kindem, J. G. Bartholomew, E. Miyazono, T. Zhong, N. Sinclair, A. Faraon, Nanophotonic quantum storage at telecommunication wavelength. Phys. Rev. Appl. 12, 024062 (2019).

[66] A. Seri, G. Corrielli, D. Lago-Rivera, A. Lenhard, H. de Riedmatten, R. Osellame, M. Mazzera, Laser-written integrated platform for quantum storage of heralded single photons. Optica 5, 934–941 (2018).

[67] X. Zhang, B. Zhang, S. Wei, H. Li, J. Liao, C. Li, G. Deng, Y. Wang, H. Song, L. You, B. Jing, F. Chen, G. Guo, Q. Zhou, Telecom-band–integrated multimode photonic quantum memory. Sci. Adv. 9, eadf4587 (2023). 37450592 10.1126/sciadv.adf4587 PMC10348679

