# Supplementary material for: A millisecond integrated quantum memory for photonic qubits
Source: Sci Adv. 2025 Mar 26;11(13):eadu5264. doi: 10.1126/sciadv.adu5264 (PMC11939051; doi:10.1126/sciadv.adu5264)
Supplement: Supplementary file 1 — Supplementary Text Figs. S1 to S5 References [file sciadv.adu5264_sm.pdf]

Science Advances
AAAS

Supplementary Materials for

A millisecond integrated quantum memory for photonic qubits

Yu-Ping Liu *et al.*

Corresponding author: Zong-Quan Zhou, zq_zhou@ustc.edu.cn; Chuan-Feng Li, cfli@ustc.edu.cn

Sci. Adv. **11**, eadu5264 (2025)
DOI: 10.1126/sciadv.adu5264

This PDF file includes:

Supplementary Text
Figs. S1 to S5
References

Supplementary Text

Additional details about the storage device

The optical waveguide constrains light through refractive index change around notches (Figure S1). The fabrication process also induces lattice structure deformation, which results in a slightly larger inhomogeneous broadening of optical transitions of Eu^{3+} ions in waveguide than that in the bulk crystal (*34, 57*). Here we measured an optical inhomogeneous broadening of 0.80 GHz and 0.65 GHz for Eu^{3+} ions inside and outside the waveguide, respectively. The peak absorption depth is 2.09 for Eu^{3+} ions inside waveguide. The spectral feature is prepared by spectral hole burning technique (Figure S2A), see (*34*) for detail. The prepared 1.8-MHz optical absorption profile is shown in Figure S2B.

The efficiency of NLPE memory can be modeled by (*34, 43*):

$$\eta = d^2 e^{-d} \left(\eta_{\text{control}}\right)^4 e^{-\gamma_{13}{}^2 t_{31}{}^2/\left(2\ln(2)/\pi^2\right)} e^{-\gamma_{\bar{3}\bar{5}}{}^2 t_{42}{}^2/\left(2\ln(2)/\pi^2\right)} e^{-2\gamma\cdot t_{42}}, \tag{S1}$$

where γ_{13} is the inhomogeneous broadening of transition $|\pm 1/2\rangle_g \rightarrow |\pm 3/2\rangle_g$, $\gamma_{\bar{3}\bar{5}}$ is the inhomogeneous broadening of transition $|3/2\rangle_{\text{e}} \rightarrow |5/2\rangle_{\text{e}}$ and γ is the effective optical decoherence rate. η_{control} is the average transfer efficiency of optical π pulses. t_{31} is the interval between the first and third optical π pulses, while t_{42} is the interval between the second and fourth optical π pulses. By measuring the echo amplitude depending on variable t_{31} and t_{42} (Figure S3A, B), we fit the parameters and get: $\gamma_{13} = 6.0 \pm 0.9$ kHz, $\gamma_{\bar{3}\bar{5}} = 18 \pm 1$ kHz, $\gamma = 8 \pm 4$ kHz, $\eta_{\text{control}} = 85\%$ (*34, 43*). The value of γ_{13} is approximately consistent with the direct measurement on spin inhomogeneous broadening using optically detected spin resonance (Figure S1E).

The quantum storage time of this device is two orders of magnitude longer than that of previous integrated quantum memories. In Figure S4, we present the storage efficiency and storage time of integrated quantum memories for light, alongside the performance of a fiber delay line, as indicated by the blue dashed line.

The storage fidelity of time-bin qubits

The fidelity of time-bin qubits is analyzed by projection on the ideal state and the orthogonal state, with photon counts of N_+ and N_-, respectively. The fidelity is then given by

$$F = \frac{N_+}{N_+ + N_-}. \tag{S2}$$

For $\mu_q = 1.07$, the measurement results for $|e\rangle$, $|l\rangle$, $|e\rangle + |l\rangle$ are provided in Figure 3A,B. Additionally, Figure S3C shows the photon-counting histogram for input qubits of $|e\rangle + i|l\rangle$.

The measured counts should include both the echo signal and the unconditional noise:

$$N_+ = (\mu_q \eta_M F_c + p_n)N; \tag{S3}$$

$$N_- = (\mu_q \eta_M (1 - F_c) + p_n)N, \tag{S4}$$

where η_M is memory efficiency, p_n is unconditional noise probability and N is experiment repetition. F_c is classical fidelity which we measured to be 99.7 %. Here we assume $F_c = 1$ for simplicity, which also gives a stricter upper limit on fidelity bound. By substituting N_+ and N_- into Equation S2, the theoretical storage fidelity can be predicted as:

$$F(\mu_q) = \frac{F_c + p_n/\mu_q\eta_M}{1 + 2p_n/\mu_q\eta_M}, \tag{S5}$$

which is indicated as the green line in Figure 3C.

According to reference (*21,58*), a classical bound on storage fidelity for weak coherent states can be derived. Since the coherent state has a Poisson distribution of $P(\mu_q, n) = \mathrm{e}^{-\mu_q}\mu_q^n/n$ with average photon number μ_q, it's possible an intercept-resend attack (*59*) can gain additional information from multi-photon incidents. The attacker can measure and then prepare a state when there's high enough photon incident $n \geqslant n_{\min}$ from the input, where $n_{\min}$ is the minimal i that matches $\left(1 - P\left(\mu_q, 0\right)\right)\eta_M - \sum_{n>i+1} P\left(\mu_q, n\right) \geq 0$. And the fidelity bond is:

$$F_{\text{classical}}(\mu_q) = \frac{\left(\frac{n_{\min}+1}{n_{\min}+2}\right)\mathit{\Gamma} + \sum\limits_{n\geqslant n_{\min}+1} \frac{n+1}{n+2} P(\mu_q, n)}{\mathit{\Gamma} + \sum\limits_{n\geqslant n_{\min}+1} P(\mu_q, n)}, \tag{S6}$$

where $\mathit{\Gamma} = \left(1 - P\left(\mu_q, 0\right)\right)\eta_M - \sum_{N>N_{\min}+1} P\left(\mu_q, n\right)$. This limit is shown with red solid line in Figure 3C.

Characterization of the coplanar electric waveguide

We have also measured the performance of coplanar electric waveguide fabricated on the D1 $\times$ b plane. With the same input RF power, the Rabi frequency was 0.8 times that of the current D2 $\times$ b configuration. Therefore, we select the latter configuration for efficient spin manipulation.

The RF field intensity correlates with the width of central electrode. We measure the π pulse length with a fixed RF pulse peak power of 4 W using spin nutation measurement (Figure S5A). For all other measurements, we use complex hyperbolic secant (CHS) pulses to generate more efficient and robust π pulses (*60, 61*).

Smaller electrodes could generate stronger magnetic fields, but the field homogeneity would degrade within the optical waveguide, leading to errors in π pulses. The size of the optical waveguide is chosen to minimize damage to the crystal and maintain a small inhomogeneous broadening. A smaller optical waveguide could achieve better RF field homogeneity, but the increased optical and spin inhomogeneous broadening could decrease NLPE efficiency. Therefore, after determining appropriate optical waveguide size, we optimize the width of the coplanar electric waveguide to achieve a balance between RF field strength and homogeneity.

To benchmark the performance of CHS π pulses for different electric waveguide widths, we detect the residual population through absorption measurements. Since the XY4 sequence is robust to pulse errors, the pulse error is estimated with a set of XX sequences similar to those in Ref. (*13*). We measure the population error after 1 to 6 XX sequences and fit for the population error per XX sequence, the resulting residual population per XX pulse for different electrode widths is shown in Figure S5A. A width of 150 μm is sufficient to achieve the required population transfer efficiency. The residual population in $|\pm 3/2\rangle_g$ tested for the electrode is 3.8 ± 0.1% after an XX sequence, which corresponds to a single-pulse error of $(0.062 \pm 0.001)\pi$ (*62*). Combined with XY4 sequence, this electric waveguide provides a satisfactory signal-to-noise ratio.

The CHS π pulse employed in actual DD has a length of 60 μs and a peak power of 4 W. As the electrodes are positioned closer to the optical waveguide, the heating effects should be evaluated. We measure the optical coherence lifetimes of Eu^{3+} ions, which is sensitive to temperature (*63*), immediately after an RF pulse excitation. Here, the RF frequency is set to 10 MHz, far off-resonance to the Eu^{3+} ions to avoid direct interaction between the RF field and the the Eu^{3+} ions and the input

peak power is raised to 6 W. According to the data shown in Figure S5B, the optical coherence lifetime is still longer than 210 μs after a 60 μs RF pulse. Taking the optical evolution time in NLPE memory into consideration, the efficiency drop due to heating effect is less than 3%. In addition, the evolution in the optical transition starts at 0.1 ms after the last CHS π pulse in NLPE-DD memory, so the device is further cooled during this interval and we deduce that the efficiency loss due to RF heating is negligible in our experiments.

We have compared the performance of XXXX and XY4 DD sequences. The $1/e$ lifetime of NLPE-DD memory is 1.6 ms for XXXX sequence and 1.9 ms for XY4 sequence, respectively (Figure S5C). In addition, XY4 sequence provides an overall higher efficiency so it is employed in the quantum storage experiments.

Meanwhile XY4 sequence provides a smaller residual population according to Ref. (*62*). The absorption difference is too small to be detected directly in the current system. Therefore, instead of directly measuring the absorption, we estimate the error of XY4 sequence by comparing the efficiency with and without applying the sequence. Since the efficiency decays with time at different rate with and without DD, we compare the fitted intercept at zero spin-wave evolving time. From the fitting in Figure S5C, the NLPE-DD memory with XY4 sequence has a zero-time intercept efficiency of 19.79 ± 0.03%, which corresponds to the maximum NLPE-DD efficiency with zero spin-wave storage time. Meanwhile, for NLPE memory of spin wave evolution without DD as shown in Figure S3B, the zero-time intercept efficiency is 19.85 ± 0.04%. Based on these two zero-time intercept efficiencies, we estimate that our device has a 99.7 ± 0.2% rephasing efficiency for spin transitions, which puts a upper bound for residual population of 0.3 ± 0.2% after an XY4 sequence.

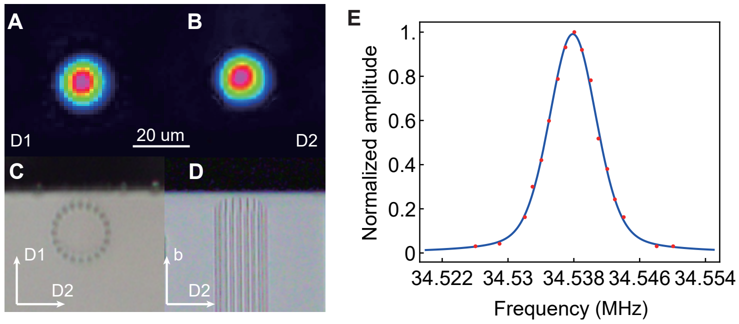

Figure S1: **Device properties.** (**A**) and (**B**) The guide mode profiles for light polarized along the D1 axis (A) and the D2 axis (B). The mode field diameters are 16.3 μm × 16.3 μm (A) and 16.2 μm × 16.5 μm (B). (**C**) and (**D**) The front view and the top view of the optical waveguide. (**E**) The inhomogeneous broadening of the hyperfine transition of Eu^{3+} ions inside the optical waveguide. The red dots represent experimental data acquired by optically detected spin nutation measurement.The blue line is the fitted curve using Voigt distribution. The full width at half maximum bandwidth of the fitted curve is 7.8 ± 0.1 kHz.

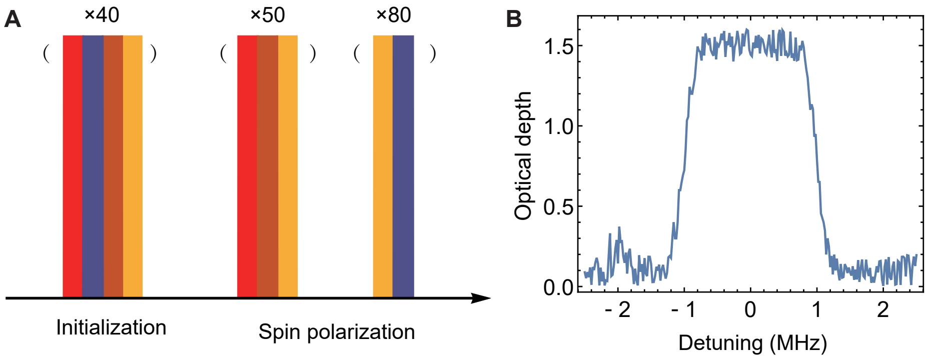

Figure S2: **Spectrum preparation.** (**A**) The preparation sequence consists of a series of 1-ms pump processes, with each pulse corresponding to a specific transition. The red pump pulse corresponds to the $|\pm 1/2\rangle_g \rightarrow |\pm 5/2\rangle_e$ transition, blue represents $|\pm 5/2\rangle_g \rightarrow |\pm 3/2\rangle_e$, brown represents $|\pm 1/2\rangle_g \rightarrow |\pm 3/2\rangle_e$ and yellow represents $|\pm 3/2\rangle_g \rightarrow |\pm 5/2\rangle_e$. (**B**) The optical absorption profile as prepared at the $|\pm 1/2\rangle_g \rightarrow |\pm 5/2\rangle_e$ transition.

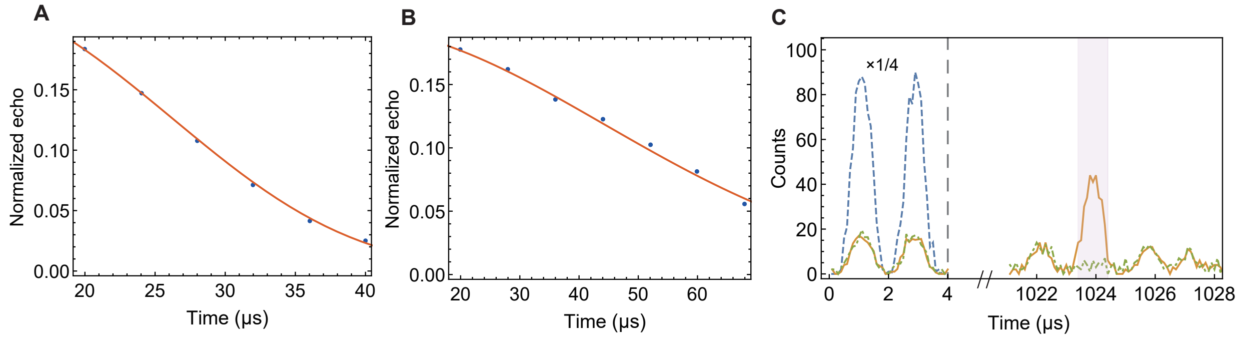

Figure S3: **NLPE memory details.** (**A**) and (**B**) Decay of NLPE echo by delaying the time interval between two π_{13} (A) and two π_{35} (B). The blue dots are normalized echo area and the red lines are fits based on Equation S1. (**C**) Photon-counting histogram for analysis of $|e\rangle + i|l\rangle$ qubit with $\mu_q = 1.07$. The inputs are shown with blue dashed line. The orange solid line and the green dash-dotted line represent the measurements with constructive and destructive interference, respectively. The light purple shaded area indicates the detection windows and the data on the left side of dashed gray line are scaled by a factor of 1/4 for visual effects.

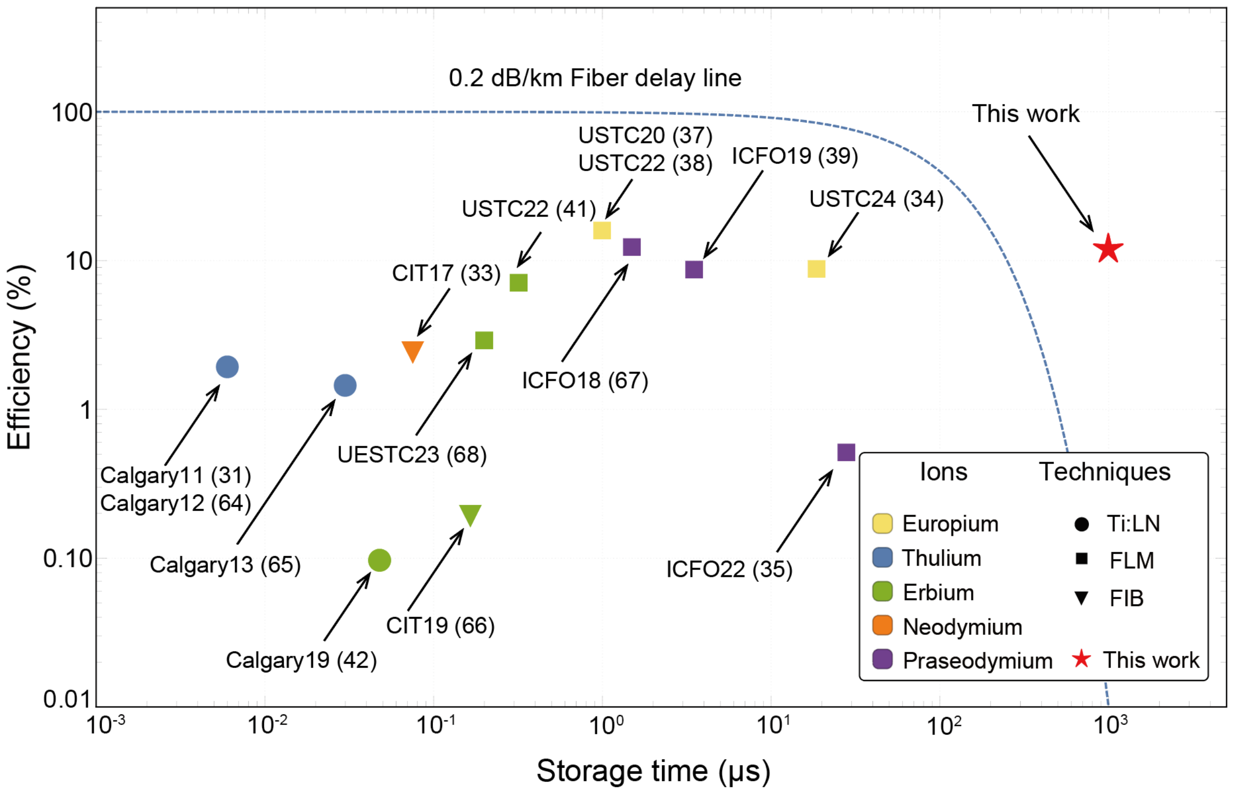

Figure S4: **The performances of integrated quantum memories for light.** The colors represent various atomic elements and the shape of dots represent various fabrication techniques, as indicated in the inset. Circular dots are Titanium-indiffused Lithium Niobate (Ti:LN) waveguides (*31, 42, 64, 65*), triangular dots are focused ion beam (FIB) milling cavities (*33, 66*), square dots are waveguides fabricated by femtosecond laser micromachining (FLM) (*34, 35, 37–39, 41, 67, 68*). The blue dashed line represents the performance of a telecom fiber delay line with a loss coefficient of 0.2 dB/km. The red star marks the current device, which is the only integrated quantum memory to outperform the fiber delay line. The data presented corresponds to the longest storage times directly demonstrated for single-photon-level quantum memory in each experiment. It is worth noting that long-duration quantum memories have also been achieved in bulk crystals (*12, 13*), though these are not included in this figure.

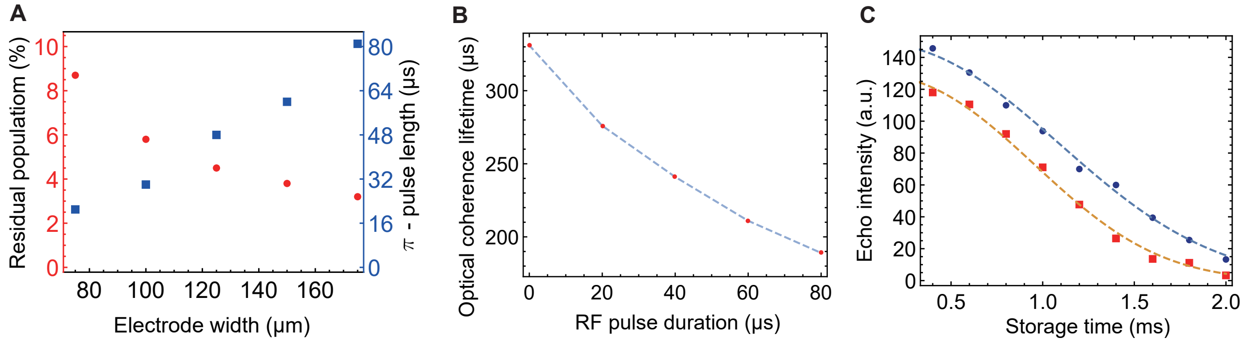

Figure S5: **Characterization of coplanar electric waveguide** (**A**) Residual population (red circular dots) per CHS XX sequence and the length of π pulses (blue squares) determined by spin nutation measurements for variable electrode width. The peak power of the RF pulses is fixed at 4 W. (**B**) Optical coherence lifetimes of Eu^{3+} measured immediately after a RF pulse excitation with variable pulse length. The peak power of the RF pulses is fixed at 6 W and the frequency is 10 MHz. (**C**) The lifetime of NLPE-DD memory. The red squares are data with a XXXX sequence and the blue circulars are data with a XY4 sequence. The lines are the corresponding fitted decay curve.

REFERENCES AND NOTES

1. H.-J. Briegel, W. Dür, J. I. Cirac, P. Zoller, Quantum repeaters: The role of imperfect local operations in quantum communication. *Phys. Rev. Lett.* **81**, 5932–5935 (1998).

2. L.-M. Duan, M. D. Lukin, J. I. Cirac, P. Zoller, Long-distance quantum communication with atomic ensembles and linear optics. *Nature* **414**, 413–418 (2001).

3. N. Sangouard, C. Simon, H. De Riedmatten, N. Gisin, Quantum repeaters based on atomic ensembles and linear optics. *Rev. Mod. Phys.* **83**, 33 (2011).

4. A. I. Lvovsky, B. C. Sanders, W. Tittel, Optical quantum memory, *Nat. Photonics* **3**, 706–714 (2009).

5. M. Gündoğan, J. S. Sidhu, V. Henderson, L. Mazzarella, J. Wolters, D. K. L. Oi, M. Krutzik, Proposal for space-borne quantum memories for global quantum networking. *npj Quantum Inf.* **7**, 128 (2021).

6. M. Gündoğan, J. S. Sidhu, M. Krutzik, D. K. L. Oi, Time-delayed single satellite quantum repeater node for global quantum communications. *Opt. Quantum* **2**, 140–147 (2024).

7. S. E. Wittig, S. M. Wittig, A. Berquanda, M. Zhong, M. Sellars, Concept for single-satellite global quantum key distribution using a solid state quantum memory, in *68th International Astronautical Congress (IAC)* (IAC-17 B, International Astronautical Federation, 2017), vol. **2**, p. 7.

8. J. Bland-Hawthorn, M. J. Sellars, J. G. Bartholomew, Quantum memories and the double-slit experiment: Implications for astronomical interferometry. *J. Opt. Soc. Am. B.* **38**, A86–A98 (2021).

9. M. Zhong, M. P. Hedges, R. L. Ahlefeldt, J. G. Bartholomew, S. E. Beavan, S. M. Wittig, J. J. Longdell, M. J. Sellars, Optically addressable nuclear spins in a solid with a six-hour coherence time. *Nature* **517**, 177–180 (2015).

10. F. Wang, M. Ren, W. Sun, M. Guo, M. J. Sellars, R. L. Ahlefeldt, J. G. Bartholomew, J. Yao, S. Liu, M. Zhong, Nuclear spins in a solid exceeding 10-hour coherence times for ultra-long-term quantum storage. *PRX Quantum* **6**, 010302 (2025).

11. Y. Ma, Y.-Z. Ma, Z.-Q. Zhou, C.-F. Li, G.-C. Guo, One-hour coherent optical storage in an atomic frequency comb memory. *Nat. Commun.* **12**, 2381 (2021).

12. A. Ortu, A. Holzäpfel, J. Etesse, M. Afzelius, Storage of photonic time-bin qubits for up to 20 ms in a rare-earth doped crystal. *npj Quantum Inf.* **8**, 29 (2022).

13. P. Jobez, C. Laplane, N. Timoney, N. Gisin, A. Ferrier, P. Goldner, M. Afzelius, Coherent spin control at the quantum level in an ensemble-based optical memory. *Phys. Rev. Lett.* **114**, 230502 (2015).

14. A. J. Stolk, K. L. van der Enden, M. C. Slater, I. te Raa-Derckx, P. Botma, J. van Rantwijk, J. J. B. Biemond, R. A. J. Hagen, R. W. Herfst, W. D. Koek, A. J. H. Meskers, R. Vollmer, E. J. van Zwet, M. Markham, A. M. Edmonds, J. F. Geus, F. Elsen, B. Jungbluth, C. Haefner, C. Tresp, J. Stuhler, S. Ritter, R. Hanson, Metropolitan-scale heralded entanglement of solid-state qubits. *Sci. Adv.* **10**, eadp6442 (2024).

15. J.-L. Liu, X. Y. Luo, Y. Yu, C.-Y. Wang, B. Wang, Y. Hu, J. Li, M.-Y. Zheng, B. Yao, Z. Yan, D. Teng, J.-W. Jiang, X.-B. Liu, X.-P. Xie, J. Zhang, Q.-H. Mao, X. Jiang, Q. Zhang, X.-H. Bao, J.-W. Pan, Creation of memory–memory entanglement in a metropolitan quantum network. *Nature* **629**, 579–585 (2024).

16. C. M. Knaut, A. Suleymanzade, Y.-C. Wei, D. R. Assumpcao, P.-J. Stas, Y. Q. Huan, B. Machielse, E. N. Knall, M. Sutula, G. Baranes, N. Sinclair, C. De-Eknamkul, D. S. Levonian, M. K. Bhaskar, H. Park, M. Lončar, M. D. Lukin, Entanglement of nanophotonic quantum memory nodes in a telecom network. *Nature* **629**, 573–578 (2024).

17. L.-M. Duan, C. Monroe *Colloquium*: Quantum networks with trapped ions *Rev. Mod. Phys.* **82**, 1209–1224 (2010).

18. V. Krutyanskiy, M. Canteri, M. Meraner, J. Bate, V. Krcmarsky, J. Schupp, N. Sangouard, B. P. Lanyon, Telecom-wavelength quantum repeater node based on a trapped-ion processor. *Phys. Rev. Lett.* **130**, 213601 (2023).

19. T. van Leent, M. Bock, F. Fertig, R. Garthoff, S. Eppelt, Y. Zhou, P. Malik, M. Seubert, T. Bauer, W. Rosenfeld, W. Zhang, C. Becher, H. Weinfurter, Entangling single atoms over 33 km telecom fibre. *Nature* **607**, 69–73 (2022).

20. B. Hensen, H. Bernien, A. E. Dréau, A. Reiserer, N. Kalb, M. S. Blok, J. Ruitenberg, R. F. Vermeulen, R. N. Schouten, C. Abellán, W. Amaya, V. Pruneri, M. W. Mitchell, M. Markham, D. J. Twitchen, D. Elkouss, S. Wehner, T. H. Taminiau, R. Hanson, Loophole-free Bell inequality violation using electron spins separated by 1.3 kilometres. *Nature* **526**, 682–686 (2015).

21. H. P. Specht, C. Nölleke, A. Reiserer, M. Uphoff, E. Figueroa, S. Ritter, G. Rempe, A single-atom quantum memory. *Nature* **473**, 190–193 (2011).

22. M. D. Eisaman, A. André, F. Massou, M. Fleischhauer, A. S. Zibrov, M. D. Lukin, Electromagnetically induced transparency with tunable single-photon pulses. *Nature* **438**, 837–841 (2005).

23. S. Ritter, C. Nölleke, C. Hahn, A. Reiserer, A. Neuzner, M. Uphoff, M. Mücke, E. Figueroa, J. Bochmann, G. Rempe, An elementary quantum network of single atoms in optical cavities. *Nature* **484**, 195–200 (2012).

24. X. Liu, J. Hu, Z. F. Li, X. Li, P. Y. Li, P. J. Liang, Z. Q. Zhou, C. F. Li, G. C. Guo, Heralded entanglement distribution between two absorptive quantum memories. *Nature* **594**, 41–45 (2021).

25. D. Lago-Rivera, S. Grandi, J. V. Rakonjac, A. Seri, H. de Riedmatten, Telecom-heralded entanglement between multimode solid-state quantum memories *Nature* **594**, 37–40 (2021).

26. H. de Riedmatten, M. Afzelius, M. U. Staudt, C. Simon, N. Gisin, A solid-state light–matter interface at the single-photon level. *Nature* **456**, 773–777 (2008).

27. M. P. Hedges, J. J. Longdell, Y. Li, M. J. Sellars, Efficient quantum memory for light. *Nature* **465**, 1052–1056 (2010).

28. T. Böttger, C. Thiel, R. Cone, Y. Sun, Effects of magnetic field orientation on optical decoherence in Er^{3+}:Y_2SiO_5. *Phys. Rev. B.Condens. Matter Mater. Phys.* **79**, 115104 (2009).

29. M. Rančić, M. P. Hedges, R. L. Ahlefeldt, M. J. Sellars, Coherence time of over a second in a telecom-compatible quantum memory storage material. *Nat. Phys.* **14**, 50–54 (2018).

30. G. Heinze, C. Hubrich, T. Halfmann, Stopped light and image storage by electromagnetically induced transparency up to the regime of one minute. *Phys. Rev. Lett.* **111**, 033601 (2013).

31. E. Saglamyurek, N Sinclair, J. Jin, J. A. Slater, D. Oblak, F. Bussières, M. George, R. Ricken, W. Sohler, W. Tittel, Broadband waveguide quantum memory for entangled photons. *Nature* **469**, 512–515 (2011).

32. E. Saglamyurek, M. Grimau Puigibert, Q. Zhou, L. Giner, F. Marsili, V. B. Verma, S. Woo Nam, L. Oesterling, D. Nippa, D. Oblak, W. Tittel, A multiplexed light-matter interface for fibre-based quantum networks. *Nat. Commun.* **7**, 11202 (2016).

33. T. Zhong J. M. Kindem, J. G. Bartholomew, J. Rochman, I. Craiciu, E. Miyazono, M. Bettinelli, E. Cavalli, V. Verma, S. W. Nam, F. Marsili, M. D. Shaw, A. D. Beyer, A. Faraon, Nanophotonic rare-earth quantum memory with optically controlled retrieval. *Science* **357**, 1392–1395 (2017).

34. T.-X. Zhu, M. X. Su, C. Liu, Y. P. Liu, C. F. Wang, P. X. Liu, Y. J. Han, Z. Q. Zhou, C. F. Li, G. C. Guo, Integrated spin-wave quantum memory. *Natl. Sci. Rev.* **11**, nwae161 (2024).

35. J. V. Rakonjac, G. Corrielli, D. Lago-Rivera, A. Seri, M. Mazzera, S. Grandi, R. Osellame, H. de Riedmatten, Storage and analysis of light-matter entanglement in a fiber-integrated system. *Sci. Adv.* **8**,eabn3919 (2022).

36. Z.-Q. Zhou, C. Liu, C.-F. Li, G.-C. Guo, D. Oblak, M. Lei, A. Faraon, M. Mazzera, H. de Riedmatten, Photonic integrated quantum memory in rare-earth doped solids. *Laser Photonics Rev.* **17**, 2300257 (2023).

37. C. Liu, T. X. Zhu, M. X. Su, Y. Z. Ma, Z. Q. Zhou, C. F. Li, G. C. Guo, On-demand quantum storage of photonic qubits in an on-chip waveguide. *Phys. Rev. Lett.* **125**, 260504 (2020).

38. T.-X. Zhu, C. Liu, M. Jin, M. X. Su, Y. P. Liu, W. J. Li, Y. Ye, Z. Q. Zhou, C. F. Li, G. C. Guo, On-demand integrated quantum memory for polarization qubits. *Phys. Rev. Lett.* **128**, 180501 (2022).

39. A. Seri, D. Lago-Rivera, A. Lenhard, G. Corrielli, R. Osellame, M. Mazzera, H. de Riedmatten, Quantum storage of frequency-multiplexed heralded single photons. *Phys. Rev. Lett.* **123**, 080502 (2019).

40. N. Sinclair, E. Saglamyurek, H. Mallahzadeh, J. A. Slater, M. George, R. Ricken, M. P. Hedges, D. Oblak, C. Simon, W. Sohler, W. Tittel, Spectral multiplexing for scalable quantum photonics using an atomic frequency comb quantum memory and feed-forward control. *Phys. Rev. Lett.* **113**, 053603 (2014).

41. D.-C. Liu, P. Y. Li, T. X. Zhu, L. Zheng, J. Y. Huang, Z. Q. Zhou, C. F. Li, G. C. Guo, On-demand storage of photonic qubits at telecom wavelengths. *Phys. Rev. Lett.* **129**, 210501 (2022).

42. M. F. Askarani, M. G. Puigibert, T. Lutz, V. B. Verma, M. D. Shaw, S. W. Nam, N. Sinclair, D. Oblak, W. Tittel, Storage and reemission of heralded telecommunication-wavelength photons using a crystal waveguide. *Phys. Rev. Appl.* **11**, 054056 (2019).

43. Y.-Z. Ma, M. Jin, D. L. Chen, Z. Q. Zhou, C. F. Li, G. C. Guo, Elimination of noise in optically rephased photon echoes. *Nat. Commun.* **12**, 4378 (2021).

44. Y. Wiemann, J. Simmendinger, C. Clauss, L. Bogani, D. Bothner, D. Koelle, R. Kleiner, M. Dressel, M. Scheffler, Observing electron spin resonance between 0.1 and 67 GHz at temperatures between 50 mK and 300 K using broadband metallic coplanar waveguides. *Appl. Phys. Lett.* **106**, 193505 (2015).

45. Y.-Z. Ma, Y.-C. Lv, T.-S. Yang, Y. Ma, Z.-Q. Zhou, C.-F. Li, G.-C. Guo, Monte Carlo simulation of the nuclear spin decoherence process in Eu^{3+}:Y_2 SiO_5 crystals. *Phys. Rev. B.* **107**, 014310 (2023).

46. P.-J. Liang, T.-X. Zhu, Y.-X. Xiao, Y.-Y. Wang, Y.-J. Han, Z.-Q. Zhou, C.-F. Li, Concentration-dependent optical and spin inhomogeneous linewidth of europium-doped yttrium orthosilicate crystals. *Acta Phys. Sin.* **73**, 100301 (2024).

47. A. G. Okhrimchuk, A. V. Shestakov, I. Khrushchev, J. Mitchell, Depressed cladding, buried waveguide laser formed in a YAG:Nd^{3+} crystal by femtosecond laser writing. *Opt. Lett.* **30**, 2248–2250 (2005).

48. N. Skryabin, A. Kalinkin, I. Dyakonov, S. Kulik, Femtosecond laser written depressed-cladding waveguide 2 × 2, 1 × 2 and 3 × 3 directional couplers in Tm^{3+}:YAG crystal. *Micromachines* **11**, 1 (2020).

49. R. N. Simons, *Coplanar Waveguide Circuits, Components, and Systems* (John Wiley & Sons, 2004).

50. C. P. Wen, Coplanar waveguide: A surface strip transmission line suitable for nonreciprocal gyromagnetic device applications. *IEEE Trans. Microw. Theory Tech.* **17**, 1087–1090 (1969).

51. Materials and methods are available as supplementary materials.

52. M. Jin, Y.-Z. Ma, Z.-Q. Zhou, C.-F. Li, G.-C. Guo, A faithful solid-state spin-wave quantum memory for polarization qubits. *Sci. Bull.* **67**, 676–678 (2022).

53. M. Gündoğan, P. M. Ledingham, K. Kutluer, M. Mazzera, H. de Riedmatten, Solid state spin-wave quantum memory for time-bin qubits. *Phys. Rev. Lett.* **114**, 230501 (2015).

54. X. Liu, X.-M. Hu, T.-X. Zhu, C. Zhang, Y.-X. Xiao, J.-L. Miao, Z.-W. Ou, P.-Y. Li, B.-H. Liu, Z.-Q. Zhou, C.-F. Li, G.-C. Guo, Distributed quantum computing over 7.0 km. *Nat. Commun.* **15**, 8529 (2024).

55. P. Jobez, I. Usmani, N. Timoney, C. Laplane, N. Gisin, M. Afzelius, Cavity-enhanced storage in an optical spin-wave memory. *New J. Phys.* **16**, 083005 (2014).

56. M. Afzelius, C. Simon, Impedance-matched cavity quantum memory. *Phys. Rev. A* **82**, 022310 (2010).

57. A. Stoneham, Shapes of inhomogeneously broadened resonance lines in solids. *Rev. Mod. Phys.* **41**, 82 (1969).

58. M. Gündoğan, P. M. Ledingham, A. Almasi, M. Cristiani, H. de Riedmatten, Quantum storage of a photonic polarization qubit in a solid. *Phys. Rev. Lett.* **108**, 190504 (2012).

59. M.Curty, N.Lütkenhaus, Intercept-resend attacks in the Bennett-Brassard 1984 quantum-key-distribution protocol with weak coherent pulses. *Phys. Rev. A*, **71**, 062301 (2005).

60. I. Roos, K. Mølmer, Quantum computing with an inhomogeneously broadened ensemble of ions: Suppression of errors from detuning variations by specially adapted pulses and coherent population trapping. *Phys. Rev. A* **69**, 022321 (2004).

61. V. Damon, M. Bonarota, A. Louchet-Chauvet, T. Chaneliere, J.-L. Le Gouët, Revival of silenced echo and quantum memory for light. *New J. Phys.* **13**, 093031 (2011).

62. E. Zambrini Cruzeiro, F. Fröwis, N. Timoney, M. Afzelius, Noise in optical quantum memories based on dynamical decoupling of spin states. *J. Mod. Opt.* **63**, 2101–2113 (2016).

63. F. Könz, Y. Sun, C. W. Thiel, R. L. Cone, R. W. Equall, R. L. Hutcheson, R. M. Macfarlane, Temperature and concentration dependence of optical dephasing, spectral-hole lifetime, and anisotropic absorption in Eu^{3+}:Y_2SiO_5. *Phys. Rev. B* **68**, 085109 (2003).

64. E. Saglamyurek, N. Sinclair, J. Jin, J. A. Slater, D. Oblak, F. Bussières, M. George, R. Ricken, W. Sohler, W. Tittel, Conditional detection of pure quantum states of light after storage in a Tm-doped waveguide. *Phys. Rev. Lett.* **108**, 083602 (2012).

65. J. Jin, J. A. Slater, E. Saglamyurek, N. Sinclair, M. George, R. Ricken, D. Oblak, W. Sohler, W. Tittel, Two-photon interference of weak coherent laser pulses recalled from separate solid-state quantum memories. *Nat. Commun.* **4**, 2386 (2013).

66. I. Craiciu, M. Lei, J. Rochman, J. M. Kindem, J. G. Bartholomew, E. Miyazono, T. Zhong, N. Sinclair, A. Faraon, Nanophotonic quantum storage at telecommunication wavelength. *Phys. Rev. Appl.* **12**, 024062 (2019).

67. A. Seri, G. Corrielli, D. Lago-Rivera, A. Lenhard, H. de Riedmatten, R. Osellame, M. Mazzera, Laser-written integrated platform for quantum storage of heralded single photons. *Optica* **5**, 934–941 (2018).

68. X. Zhang, B. Zhang, S. Wei, H. Li, J. Liao, C. Li, G. Deng, Y. Wang, H. Song, L. You, B. Jing, F. Chen, G. Guo, Q. Zhou, Telecom-band–integrated multimode photonic quantum memory. *Sci. Adv.* **9**, eadf4587 (2023).
